# Autoimmune Heparin-Induced Thrombocytopenia after Transcatheter Aortic Valve Implantation: Successful Treatment with Adjunct High-Dose Intravenous Immunoglobulin

**DOI:** 10.1055/s-0039-1692990

**Published:** 2019-06-28

**Authors:** Tamam Bakchoul, Oliver Borst, Reimer Riessen, Josip Lucic, Meinrad Gawaz, Karina Althaus, Parwez Aidery

**Affiliations:** 1 Transfusion Medicine, University Hospital of Tübingen, Tübingen, Germany; 2 Department of Cardiology and Cardiovascular Medicine, University Hospital of Tübingen, Tübingen, Germany; 3 Department of Internal Medicine, Medical Intensive Care Unit, University of Tübingen, Tübingen, Germany

**Keywords:** thrombocytopenia, heparin, antibody

## Abstract

We describe a rare case of autoimmune heparin-induced thrombocytopenia after transcatheter aortic valve implantation in which antibodies against platelet factor 4/heparin have led to platelet activation even after heparin cessation, causing a delayed drop in platelet count to below 20 × 10 ^9^ /L. Most interestingly, platelet count rapidly improved after intravenous immunoglobulin treatment and no new thromboembolic complications were observed with further anticoagulation with rivaroxaban.

Autoimmune heparin-induced thrombocytopenia (aHIT) is a subset of HIT in which patients have high levels of antiplatelet factor 4 (PF4)/heparin antibodies that are able to activate platelets even in the absence of heparin. 1 2 3 4 Due to the autoimmune nature of aHIT, discontinuation of heparin alone is not adequate and patients should be promptly treated with therapeutic doses of a nonheparin anticoagulant. Normalization of platelet counts upon start of alternative anticoagulation in aHIT, however, delays for several days and thrombocytopenia may persist. 1 5 In certain situations, a rapid recovery in platelet count is needed such as in patients who require antiplatelet treatment (APT). In this article, we report on the use of intravenous immunoglobulin (IVIG) as successful adjunctive treatment of aHIT after transcatheter aortic valve implantation (TAVI).

A 68-year-old male patient (96 kg, 160 cm) with a 12-year history of diabetes mellitus, arterial hypertension, and chronic renal insufficiency was admitted to our hospital with cardiac decompensation and cardiogenic shock due to aortic valve stenosis. The patient underwent coronary angiography which revealed severe calcification of the valve that caused reduced left ventricular (LV) function with an ejection fraction of 30%. An emergency percutaneous transluminal balloon valvuloplasty (PTV) was performed after giving one dose of low-molecular-weight heparin (LMWH; enoxaparin, 4,000 anti-Xa units, s.c.). Cardiogenic shock persists despite PTV, and progressive worsening of LV function with increasing catecholamine requirement was observed. Therefore, a transcatheter aortic valve was implemented via a transfemoral approach. For the TAVI procedure, another 4,000 anti-Xa units of LMWH (enoxaparin) was given. Additionally, patient received dual-antiplatelet treatment (DAPT) consisting of aspirin (ASA 100 mg/day) and clopidogrel (Plavix 75 mg/day). The TAVI procedure was uneventful and patient was successfully weaned and extubated 48 hours after intervention.

On day 7, platelet count dropped to 98 × 10 ^9^ /L ( Fig. 1 ) and thrombocytopenia was initially thought to be due to platelet consumption. The next day, a further drop in platelet count to below 30 × 10 ^9^ /L was documented and DAPT was discontinued to avoid potential bleeding. The pretest probability for HIT was estimated using the 4Ts score revealing a score of 5. Strong anti-PF4/heparin immunoglobulin G antibodies were detected in patient's serum using enzyme-linked immunosorbent assay (optical density: 3.825, cutoff: 0.300). Platelet activation was investigated using the functional assay HIPA. Patient's serum induced rapid activation of platelets from four donors in the presence of 0.2 IU/mL LMWH (reviparin) as well as in the absence of heparin, but not after addition of high concentration of heparin (100 IU/mL). Duplex ultrasound imaging of the lower-limb veins confirmed subclinical deep-vein thrombosis (left superficial femoral vein).

**Fig. 1:**
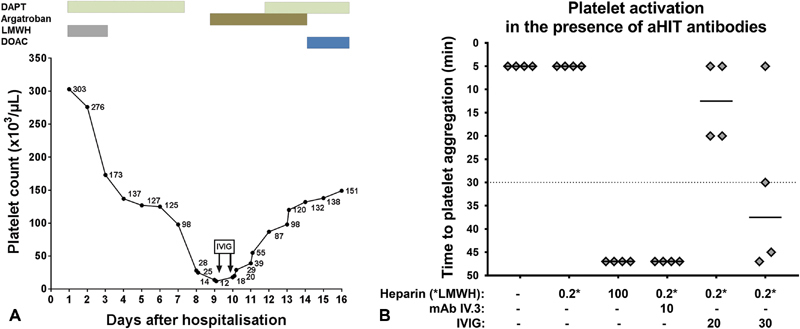
( **A** ) Recovery in platelet count in an autoimmune HIT after transcatheter aortic valve. **(B)** The impact of IVIG on platelet activation by autoimmune HIT antibodies. Patient's serum was incubated with platelets from four healthy donors in the presence of 0.2 IU/mL LMWH (reviparin) and different concentrations of IVIG. Platelet activation was determined by measurement of time until platelet aggregation in the HIPA assay. DAPT, dual antiplatelet therapy; DOAC, direct oral anticoagulant; HIT, heparin-induced thrombocytopenia; IVIG, intravenous immunoglobulin; LMWH, low-molecular-weight heparin; MAb IV.3, Fc gamma receptor IIa blocking monoclonal antibody.

Owing to the recent TAVI, rapid correction of platelet count to values higher than 50 × 10 ^9^ /L was required to enable prompt start of DAPT in addition to the anticoagulation (HIT-associated thrombosis). Recent studies showed that high-dose IVIG can inhibit platelet activation in HIT indicating an additional treatment option in patients with aHIT. 6 7 8 To verify the inhibitory effect of IVIG, the HIPA was performed in the presence of patient's serum, 0.2 IU/mL LMWH and IVIG. A strong inhibition of platelet activation was observed at a final concentration of 30 mg/mL IVIG ( Fig. 1 ). Our patient was then treated with a combination of argatroban (2 µg/kg body weight per minute, Argatra) and IVIG (100 g/day for 2 consecutive days, Gamunex 10%). Platelet count increased within 2 days to reach values higher than 50 × 10 ^9^ /L, where we felt comfortable to restart DAPT ( Fig. 1 ). The patient was switched to rivaroxaban as platelet count recovered and discharged on day 16. After 3 months, a follow-up testing revealed normal platelet count and no new thrombotic events under rivaroxaban.

To our knowledge, this is the first case of an aHIT after TAVI. This severe form of HIT was caused by anti-PF4 antibodies that are able to activate platelets even in the absence of heparin. As in our patient, recent case reports indicated that high-dose IVIG can inhibit platelet activation by HIT antibodies and accelerate platelet count recovery in aHIT patients. 7 8 However, it should be emphasized that due to the known thrombotic risk of high-dose IVIG treatment, this therapeutic option should be used with caution in the prothrombotic disease aHIT. In fact, it was recently suggested that patients with aHIT who are treated with high-dose IVIG should undergo serial measurements of hemostatic markers, including D-dimer, to exclude thrombotic side effects. 8

Current guidelines recommend the use of aspirin plus ADP receptor blocker for the initial 1 to 6 months following the TAVI procedure. 9 10 In addition, recent data showed that anticoagulation decreases the risk of bioprosthetic valve dysfunction after TAVI. 11 However, in thrombocytopenic patients, triple antithrombotic treatment is associated with increased risk of bleeding. 12 Therefore, we believed it was important to raise the platelet count quickly so as to be able to administer triple antithrombotic therapy in this high-risk post-TAVI patient. In addition, taking into consideration the HIT-associated thrombosis, we decided to combine argatroban treatment with high-dose IVIG which successfully increased platelet count to above 50 × 10 ^9^ /L enabling a safe restart of APT.

For long-term antithrombotic treatment, emerging evidence suggests that direct oral anticoagulants (DOACs) can be used in HIT. 6 However, published experience with these drugs in aHIT patients is still limited and does not allow final conclusion on their safety and efficacy to treat this severe form of HIT. Of a particular importance appears to be the observed low trough levels of the DOAC, which might cause inadequate protection against new thrombosis in aHIT patients. In our case, we started the rivaroxaban treatment as platelet count recovered to stabile values indicating normal hemostasis. A follow-up testing after 3 months revealed no new thrombotic events. Consistent with previous report, 13 our finding suggests that rivaroxaban may be safely used to prevent new thrombosis in aHIT patients.

Taken together, our case shows that the management of HIT after TAVI is challenging as patients need immediate DAPT treatment to avoid cardiovascular complications. 12 A combined administration of nonheparin anticoagulation and IVIG was effective to achieve safe platelet count that enabled rapid restart of DAPT. Our observation supports the emerging concept that high-dose IVIG is effective for treating aHIT disorders including delay onset of HIT syndrome.
